# Repeated cardiopulmonary performance measurements in young competitive handball players with and without SARS-CoV-2 infection

**DOI:** 10.1186/s13102-022-00591-2

**Published:** 2022-11-23

**Authors:** Johannes Lässing, S. Kwast, C. Bischoff, N. Hölldobler, M. Vondran, R. Falz, M. Busse

**Affiliations:** 1 Institute of Exercise Science & Sports Medicine, Martin Luther University Halle-Wittenberg, Von-Seckendorff-Platz 2, 06120 Halle (Saale), Germany; 2 Institute of Sport Medicine and Prevention, University of Leipzig, Leipzig, Germany; 3 Department of Cardiovascular Surgery, Philipps-University Marburg, University Hospital Gießen and Marburg, Marburg, Germany

**Keywords:** Reduced stroke volume, Diffusion capacity, Covid-19, Reduced exercise capacity, Arrhythmia

## Abstract

**Background:**

The SARS-CoV-2 virus and its long-term consequences in adolescents have a global impact on upcoming medical issues. The aim of this study was to investigate the effects of a SARS-CoV-2 infection on cardiorespiratory parameters in young athletes.

**Methods:**

In a cohort study involving repeated measurements during a six-month period, cardiorespiratory parameters were assessed in infected (SCoV) and non-infected (noSCoV) athletes. We evaluated handball players (17.2 ± 1.0 years) via performance diagnostics and a specific examination after a SARS-CoV-2 infection or without.

**Results:**

We observed no significant differences between the two groups at the first visit*.* But between the first and second visit, the SCoV group’s maximum power output was significantly lower than the noSCoV group’s (− 48.3 ± 12.5; *p* ≤ 0.01 vs. − 15.0 ± 26.0 W; *p* = 0.09). At the second visit, lung diffusion capacity (DL_CO_/V_A_, %predicted) did not differ between groups (111.6 ± 11.5 vs. 116.1 ± 11.8%; *p* = 0.45). HR during comparative stress showed no group differences. The SCoV group’s mean oxygen uptake during incremental exercise was lower (Two-way-ANOVA: 1912 vs. 2106 ml; *p* ≤ 0.01; mean difference: − 194 ml; 95% CI − 317 to − 71); we also noted a significantly lower stroke volume course during exercise (Two-way-ANAOVA: 147.5 vs. 169.5 ml; mean difference: − 22 ml; *p* ≤ 0.01; 95% CI − 34.2 to − 9.9). The probability of premature ventricular complexes after a SARS-CoV-2 infection yielded an odds ratio of 1.6 (95% CI 0.24–10.81).

**Conclusions:**

The physical performance of young athletes infected with SARS-CoV-2 was impaired. This decreased performance is probably due to cardiac and/or peripheral deconditioning. Studies with larger cohorts are needed to make more profound conclusions.

## Introduction

Comprehensive studies have shown that sudden cardiac death in athletes had a pre-Corona pandemic incidence of 1:50,000 athlete-years [1]. There is evidence that myocarditis was responsible for 3.8% of sudden cardiac deaths in athletes before the SARS-CoV-2 virus was discovered [2]. The effects of SARS-VoV-2 infection and its consequences for competitive athletes are not fully established. There is an urgent need to develop recommendations for their safe return to physical activity [3, 4]. Daniels et al. [5] reported that myocarditis was diagnosed in 2.3% of 1597 US competitive athletes from various disciplines studied. The course of Covid-19 disease in young healthy competitive athletes is usually mild or asymptomatic, and independent from the sport category [6]. Numerous studies have shown that even athletes with a mild infection course can present cardiac abnormalities such as myocarditis after SARS-CoV-2 infection [5–8]. A recent review of a SARS-CoV-2 infection’s effects reported the likelihood of cardiovascular deconditioning with the consequences of subnormal cardiac performance in the form of reduced stroke volume and lower VO_2_ peak values [9]. …(this segment we have moved to the discussion)… Other authors have reported contradictory findings such as a substantially lower prevalence of diagnosed cardiac events in competitive athletes after an asymptomatic or mild infection course [3, 10–12]. The latest evidence thus indicates predominantly less frequent cardiac pathologies in athletes with a mainly mild course, but also reveals abnormal cardiac findings independent of symptoms [5, 8, 13, 14]. There is thus an urgent need for targeted screening to ensure these athletes’ return to a high level of physical activity [3, 5, 7, 8, 10].

In sum, despite a Covid-19 infection’s less symptomatic presentation in young athletes, cardiac involvement consistent with clinical or subclinical myocarditis seems possible [5, 7, 15]. However, myocardial impairment can in some cases lead to cardiac dysfunction such as electrocardiogram (ECG) arrhythmias or echocardiographic abnormalities [3, 15, 16]. Echocardiographic diagnoses in conjunction with assessing global longitudinal strain (GLS) may be a good marker of inflammatory myocarditis, even in the presence of an unchanged ejection fraction [17, 18]. Otherwise, although elite athletes showed no GLS impairments, they did demonstrate a decrease in oxygen uptake (V̇O_2_), pulse rate and respiratory minute volume on an ergometer test after SARS-CoV-2 infection [13]. Mitrani et al. [12] showed that athletes with proven heart abnormalities had ventricular extra beats and/or reduced ventilatory efficiency (VE/VCO_2_) without GLS or V̇O_2_ differences compared with their group with no myocardial abnormalities.

Objectified performance limitations in terms of hemodynamic, cardiopulmonary, and metabolic performance are currently poorly known. For these reasons, the present work compares repeated exercise data from a team of competitive adolescent handball players with and without SARS-CoV-2 infection (9 of 18 players with infection). Our study aim was to examine potential physiological abnormalities in young, supposedly healthy, competitive athletes following a SARS-CoV-2 infection. Because of deconditioning effects after a SARS-CoV-2 infection, performance impairments in the SCoV-group would be likely.

## Materials and methods

### Ethics approval and study group

The examinations were carried out within the context of a cooperation agreement with the SC DHfK Handball Leipzig. This collaboration includes providing medical care to the 18 listed young handball players (age: 17.2 ± 1.0 years; height: 186.6 ± 7.0 cm; weight: 80.6 ± 11.7 kg), verifying their physical fitness for competition. After being given verbal and written information, all participants provided their written informed consent.

**Fig. 1:**
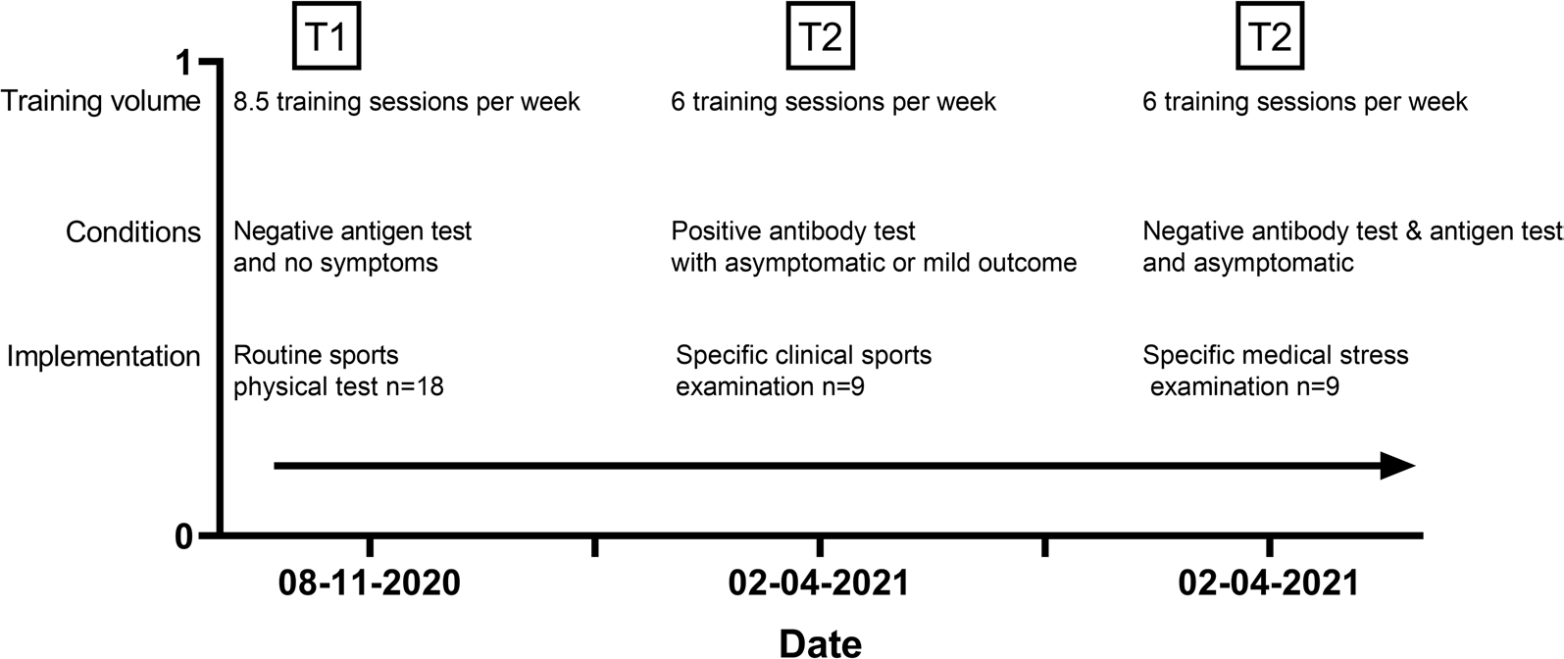
Examinations from 08.2020 to 04.2021 and pandemic-induced training intensities

Figure 1 illustrates the examination schedules of sports fitness tests and clinical checkups of handball players with and without a SARS-CoV-2 infection.

From August to October 2020, these handball players were screened during a routine sport medical exercise fitness examination (pre-test T1), for the upcoming season (2020/2021). None of the handball players we examined tested positive in the rapid antigen test (COVID-19 Ag RAPID TEST DEVICE, Abbott Rapid Diagnostics Jena GmbH, Germany) or demonstrated impaired fitness for exercise. None of the players reported having had an infectious disease in the previous months. Additionally, all players were tested several times per week during this period at school: via a rapid antigen test at the sports boarding school and during each training session (Clungene COVID-19 Antigen Rapid Test, Hangzhou Clongene Biotechnology Co., Ltd) due to government regulations and to minimize infection-related absence. In the period up to February 2021, nine players tested positive for the SARS-CoV-2-Virus in a rapid antigen test, and those results were confirmed via a transcription polymerase chain reaction (RT-PCR) [19] test. From February 2021 to April 2021 the 18 handball players were re-examined by undergoing the medical exercise fitness examination (post-test T2) targeting specific clinical aspects. Laboratory antibody testing (Serum, SARS-CoV-2 IgG antibodies) confirmed that 9 handball players had been infected with SARS-CoV-2 virus (SCoV) and the 9 other players presented no antibodies to SARS-CoV-2 virus or positive antigen test results (noSCoV). A recently published study found that these nucleoprotein-based tests can reliably detect antibody levels for up to at least 120 days [20]. Thus, no player in the noSCoV group ever tested positive through rapid antigen tests and none had any antibodies in the serologic finding at the second testing time point (T2). In the SCoV group (SCoV, n = 9), only two had suffered symptoms such as the loss of taste and smell. None of the 18 handball players was vaccinated at that time. Table 1 contains data on all athletes included in this study.

**Table 1:** Subject information and symptom reporting

	Mean ± (SD) SCoV	Mean ± SD noSCoV
N =	9	9
Age (years)	17.1 ± 1.2	17.1 ± 0.90
Weight (kg)	78.6 ± 12.1	81.6 ± 11.9
Height (cm)	184.9 ± 5.4	186.8 ± 7.5
Time from pre-test (T1) to post-test (T2) (days)	226.6 ± 27.1	245.3 ± 12.6
N = 8 Time from positive COVID-19 test result to clinical tests (days)	28.3 ± 14.9	–
*Clinical symptoms*		
No symptoms	6	9
Headache	0	0
Rhinitis	0	0
No sense of taste	2	0
No sense of smell	2	0
Chest pain	0	0
Breathing problems	0	0
Sore throat, cough, sniffles	1	0
Listlessness	1	0

Subject information from all athletes included in this study, N = Number of cases, T1 = first examination, T2 = second examination

### Study design

This listed cohort study involves two study time points (pre T1 and post T2). Compared were an incremental exercise test (pre-test T1) with no detectable SARS-CoV-2 infection for all 18 athletes, and a specific stress test (post-test T2) in which 9 subjects had been verifiably infected with SARS-CoV-2 and 9 subjects showed no evidence of an expired infection.

*Routine examinations at baseline (T1)*: Exercise tolerance was tested on two days. On the first day, informed consent for the exercise examination was obtained from the players or their legal guardians. Laboratory chemical blood tests were normal (small blood count, C-reactive protein). After assessing their laboratory results, the players were examined for the presence of SARS-CoV-2 infection on the second day via a rapid antigen test as well as a special symptom and signs questionnaire to classify the risk for the presence of COVID-19 (COVID-19 during patient admission: information, determination of risk, Thieme Compliance GmbH [Dok30a], Germany). This was followed by pulmonary function tests (Spirometer easy on PC, ndd Medizintechnik, Switzerland), echocardiography (Vivid E9- or Vivid E95, GE Healthcare Ultrasound, Horten, Norway), and an incremental cycle ergometer test with electrocardiogram (Custo cardio 300 BT_A, custo med GmbH, Germany) and manually measuring blood pressure.

*Examinations after infection (T2)*: The group of SCoV (n = 9) and noSCoV (n = 9) underwent the study visit 2 on three days as part of clinical testing for resuming sports activity. On the first day, we recorded their medical history and ran a rapid antigen test. With their consent, a blood test was done again that was expanded to include a SARS-CoV-2 antibody test (Serum, SARS-CoV-2 IgG) and to rule out acute infectious events (C-reactive protein). Cardiac laboratory markers were also analyzed (Creatine kinase MB, highly sensitive Troponin T, NT-proBNP).

The second examination day included a medical examination and questionnaire (sporting activity, smoking and alcohol consumption). They then underwent echocardiography, measurement of the lung transfer factor for carbon monoxide ([DLCO/ProMED pul-d He/CO_M_ 9.3%], EasyOne Pro, ndd Medizintechnik AG, Switzerland) and body plethysmography (ZAN500 Body, nSpire Health GmbH, Germany). At the end of the second test day, the subjects were fitted with a mobile long-range blood pressure monitor (TM-2450 long-term blood pressure monitor, BOSCH + SOHN GmbH u. Co. KG, Germany) that measures blood pressure over a 24-h period (measurement during the day until 10 p.m., every 15 min, and from 10 p.m. until 6 a.m., every 30 min). On the third measurement day, we ran another rapid antigen test and they underwent an incremental cycle ergometer test.

### Measurements

*Lung function*: PC spirometry (TrueFlow™ Technologie, ndd Medizintechnik AG, Zürich) and body plethysmography (ZAN500 Body, nSpire Health GmbH, Germany) were carried out. PC spirometry of the pretest (T1) was collected based on statically measured vital capacity (VC) as well as dynamic measurements of forced expiratory volume (FEV_1_) and peak flow (PEF) during dynamic measurements. In the post-test (T2), body plethysmography was measured in conjunction with dynamic and static lung function parameters and airway resistance (RAW).

*Incremental cycle ergometer test*: The incremental exercise was done to assess clinical and exercise physiological parameters on both test dates (T1 and T2) according to the identical ergometric protocol.

The test participants started with 50 W, which increased by 15 W min^−1^ until the maximum load was reached. This was followed by a 5-min active recovery phase during which 25% of the maximum power was used. All tests were conducted on a semi-recumbent revolution independent cycle ergometer (Ergometrics 900, ergoline GmbH, Bitz, Germany) at 70 revolutions per minute.

*At visit 1 (T1)* blood pressure was measured every 3 min during exercise, at maximum load and during the 3 min after exercise, and we asked the athletes to rate their perceived exertion (RPE). An electrocardiogram (Custo cardio 300 BT_A, custo med GmbH, Germany or Ergo script EK 3012, Ergo-line GmbH, Germany) was recorded continuously under rest, exercise, and active recovery conditions.

*At visit 2 (T2), the second visit after infection (9 of 18 participants were infected)*, Spirometry (Dynostics, Sicada GmbH, Germany), thoracic impedance (PhysioFlow, Manatec Biomedical, France), and an electrocardiogram (Cardiac PC-EKG, MESA Medizintechnik GmbH, Deutschland) were synchronized and recorded simultaneously during the whole examination. Blood lactate samples (20 µl) (Super GL, ISO 7550, Germany), blood pressure (BP) and the RPE (from 1 to 10, if 10 was total exhaustion) were assessed every 3 min, at maximum load and during the 3 min after exercise.

*Echocardiography*: The athletes underwent our standardized clinical 2D transthoracic echocardiography (Vivid E9- or Vivid E95, GE Healthcare Ultrasound, Horten, Norway) protocol. Global strain (GLS) was analyzed by 2D speckle tracking echocardiography. The ejection fraction was determined via the Teich method.

### Calculations

To determine differences within groups (noSCoV vs. SCoV) between the two measurement time points (T1 and T2), mean values of heart rate (HR), systolic blood pressure (SBP), diastolic blood pressure (DBP), rating of perceived exertion (RPE), and rate pressure product (RPP = HR × SBP) were compared during maximum stress.

For the post-specific analysis (T2) of potential physiological regulation differences and to differentiate limitations between heart, lungs and metabolism in connection with SARS-CoV-2 infection, we monitored cardiac output (CO), stroke volume (SV) and heart rate (HR); maximum oxygen consumption (V̇O_2_ max), and respiratory parameters (minute ventilation [V_E_], tidal volume [V_T_], respiratory rate [RR]) continuously at rest, during exercise, and during recovery. Averaged data were presented as a percentage of the maximum wattage level achieved at 0%, 25%, 50%, 85%, and 100% and three minutes of active recovery time (25%). We compared the physiological parameters of subjects under identical absolute wattage conditions at the same cycled 125-W stage. The stress data determined manually were taken from the exercise test protocol. Blood samples of 20 µl were drawn from the earlobe and analyzed immediately via the enzymatic-amperometric method (Super GL, Dr. Mueller Geraetebau GmbH, Freital, Germany). Stroke work (SW) was measured in Joules (J) and calculated according to the formula SW = SV × MAP/7.5 [21].

### Statistical analyses

All values are presented as means with standard deviation and the significance level was defined as *p* < 0.05. GraphPad Prism 8 (GraphPad Software Inc., California, USA) was used for statistical analysis and figure generation.

Changes within groups and between groups at pre-test and post-test (Table 2) were subjected to two-way ANOVA with repeated measures (main effects: group, time and interaction) followed by pairwise comparison with Bonferroni´s multiple comparisons test for time effects (Table 2). For distribution analysis, the Kolmogorov–Smirnov normality test was used. Sphericity was determined based on the epsilon value of the Geisser greenhouse (ℇ). If the sphericity was rejected, Greenhouse Geisser correction would apply.

**Table 2:** Changes within groups between the pre-test (T1) and post-test (T2) (ANOVA with repeated measures; post-hoc tests [pre vs. post])

	SCoV			noSCoV			Main effects (group, time, interaction)		
	T1	T2	*p*-value T1 versus T2	T1	T2	*p*-value T1 versus T2	Group F-value; *p*-value	Time F-value; *p*-value	Interaction F-value; *p*-value
*Clinical parameters*									
EF% (Teich)	62.4 ± 2.8	62.4 ± 4.8	> 0.99	62.4 ± 3.8	64.1 ± 4.7	0.56	F = 0.26 *p* = 0.62	F = 0.63 *p* = 0.44	F = 0.63 *p* = 0.44
GLS%	− 19.5 ± 3.4	− 17.0 ± 1.7	< 0.01*	− 18.4 ± 2.1	− 16.5 ± 1.7	0.03*	F = 0.97 *p* = 0.34	F = 19.4 *p* ≤ 0.01	F = 0.15 *p* = 0.71
hsCRP (mg/l)	0.5 ± 0.3	0.7 ± 0.2	0.16	0.5 ± 0.3	0.6 ± 0.3	0.52	F = 0.38 *p* = 0.55	F = 4.7 *p* ≤ 0.05*	F = 0.25 *p* = 0.63
*Pulmonary parameters*									
VC (l)	5.76 ± 0.53	5.78 ± 0.53	> 0.99	5.51. ± 0.73	5.71 ± 0.55	0.50	F = 0.39 *p* = 0.54	F = 0.84 *p* = 0.37	F = 0.59 *p* = 0.46
FEV1 (l)	4.82 ± 0.48	4.79 ± 0.62	> 0.99	4.92 ± 0.46	4.98 ± 0.55	> 0.99	F = 0.35 *p* = 0.56	F = 0.04 *p* = 0.84	F = 0.38 *p* = 0.55
PEF (l·s − 1)	9.40 ± 1.30	9.78 ± 0.83	0.90	9.51 ± 1.27	10.50 ± 1.79	0.11	F = 0.61 *p* = 0.45	F = 4.0 *p* = 0.06	F = 82 *p* = 0.38
*Exercise parameters*									
Maximum Power (W)	306.7 ± 38.6	258.30 ± 34.7	< 0.01*	300.0 ± 32.7	285.0 ± 38.2	0.09	F = 0.37/*p* = 0.55	F = 43.4 *p* ≤ 0.01*	F = 12.0 *p* ≤ 0.01*
SBP (mmHg)	202.8 ± 25.6	218.9 ± 21.0	0.10	214.4 ± 18.6	226.1 ± 22.1	0.29	F = 1.1 *p* = 0.30	F = 6.7 *p* = 0.02*	F = 0.17 *p* = 68
DBP (mmHg)	75.0 ± 10.3	80.6 ± 3.9	0.36	78.9 ± 86.1	86.1 ± 6.1	0.18	F = 3.7 *p* = 0.07	F = 5.1 *p* = 0.04*	F = 0.09 *p* = 7.7
HR (bpm)	179.4 ± 10.4	174.5 ± 4.7	0.28	184.2 ± 9.9	178.8 ± 11.1	0.21	F = 1.4 *p* = 0.25	F = 5.4 *p* = 0.03*	F = 0.01 *p* = 0.91
RPP	362.6 ± 38.5	381.9 ± 36.6	0.44	394.0 ± 28.2	404.4 ± 47.2	> 0.99	F = 3.5 *p* = 0.08	F = 1.9 *p* = 0.19	F = 0.17 *p* = 0.68
RPE	10.0 ± 0.0	9.1 ± 0.8	< 0.01*	9.9 ± 0.5	9.6 ± 0.5	0.29	F = 0.88 *p* = 0.36	F = 15.6 *p* ≤ 0.01*	F = 3.3 *p* = 0.09

Pre-test [T1]–post-test [T2], SCoV, group with SARS-CoV-2; noSCoV, group without SARS-CoV-2; FEV1, forced expiratory volume in 1 s, PEF, peak expiratory flow; VC, vital capacity; SBP, systolic blood pressure (3-min interval); DBP, diastolic blood pressure (3-min interval); RPE, rating of perceived exertion; RPP, rate pressure product; HR, heart rate; EF%, ejection fraction; GLS%, global longitudinal strain; hsCRP mg/l, high-sensitivity C-reactive protein

*Significant differences, the values are given as the means and standard deviations. No differences in post hoc tests for group differences were noted

For the specific diagnostic analysis at post-test (Tables 3, 4), rest values (lung function, echocardiography, laboratory parameters and 24 h blood pressure) and exercise values at the 125-W stage and at peak power were analyzed for normal distribution. An unpaired *t*-test was performed for these independent group comparisons if a normal distribution was present; if not, the Mann–Whitney test was used (Tables 3, 4). For the parametric test, the effect size partial eta squared n^2^_p_ was also presented.

**Table 3:** Results of the diagnostic examination DLCO, blood pressure 24 h-monitoring and echocardiography at post-test T2 between groups (unpaired *t*-test)

Parameters in T2	SCoV Mean ± SD	noSCoV Mean ± SD	*p*-value	n^2^_p_
*Resting parameters*				
R_AW_ (kPa l^−1^)	0.28 ± 0.06	0.25 ± 0.06	0.23	0.09
DL_CO_, %predicted	124.3 ± 13.7	113.8 ± 13.1	0.14	0.15
DL_CO_/V_A_, %predicted	111.6 ± 11.5	116.1 ± 11.8	0.45	0.04
24 h SBP (mmHg)	137.5 ± 12.4	134.8 ± 7.0	0.61	0.02
24 h DBP (mmHg)	76.3 ± 6.4	75.6 ± 7.0	0.80	0.01
EF% (Teich)	62.4 ± 4.8	64.1 ± 4.7	0.47	0.03
GLS%	− 16.6 ± 2.0	− 16.5 ± 1.7	0.88	< 0.01
hsCRP (mg/l)	0.68 ± 0.2	0.60 ± 0.0	0.17	0.13

SCoV, group with SARS-CoV-2; noSCoV, group without SARS-CoV-2; R_AW_, airway resistance; DL_CO_ %predicted, %predicted standard single-breath lung diffusing capacity for carbon monoxide; DL_CO_/VA %predicted, %predicted diffusing capacity divided by the alveolar volume; 24 h SBP (mmHg), mean systolic blood pressure over a 24-h period; 24 h DBP (mmHg), mean diastolic blood pressure over a 24-h period; n^2^_p_, partial eta-squared; EF%, Ejection fraction; GLS%, global longitudinal strain; hsCRP (mg/l), high-sensitivity C-reactive protein

*Significant differences, the values are given as the means and standard deviations

**Table 4:** Changes between groups at post-test T2 at 125 W and peak power (unpaired *t*-test)

Parameters	SCoV		noSCoV		*p*-value 125 W	n^2^_p_	*p*-value peak power	n^2^_p_
	125 W	Peak power	125 W	Peak power				
SBP (mmHg)	164.4 ± 12.1	218.9 ± 21.0	168.9 ± 15.2	226.1 ± 22.1	0.50	0.03	0.49	0.03
DBP (mmHg)	75.6 ± 7.7	80.6 ± 3.9	79.4 ± 5.3	86.1 ± 6.0	0.23	0.10	0.03*	0.25
HR (bpm)	122.9 ± 11.6	174.5 ± 4.7	124.0 ± 10.8	178.8 ± 11.1	0.84	< 0.01	0.30	0.07
SV (ml)	141.4 ± 22.2	162.8 ± 27.9	164.2 ± 35.6	186.8 ± 40.2	0.13	0.17	0.16	0.13
SW (J)	2.1 ± 0.4	3.1 ± 0.7	2.6 ± 0.6	3.7 ± 0.9	0.10	0.17	0.11	0.16
V_E_ (l·min^−1^)	44.9 ± 6.0	106.2 ± 13.4	49.7 ± 6.8	129.7 ± 19.8	0.13	0.14	< 0.01*	0.35
V̇O_2_ (ml·min)	1834 ± 238	3316 ± 438	1977 ± 332	3604 ± 308	0.31	0.07	0.13	0.15
LAC (mmol·l)	1.5 ± 0.6	6.8 ± 1.2	1.8 ± 0.7	7.5 ± 1.5	0.26	0.08	0.30	0.07
RPE	3.0 ± 0.9	9.1 ± 0.8	3.0 ± 0.9	9.6 ± 0.5	> 0.99	< 0.01	0.18	0.11
Power output (W)	125	258.3 ± 34.7	125	285.0 ± 38.2	–	–	0.14	0.13

Peak power, mean values maximum wattage; SCoV, group with SARS-CoV-2; noSCoV, group without SARS-CoV-2; SBP, systolic blood pressure (3-min interval); DBP, diastolic blood pressure; RPE, rating of perceived exertion; HR, heart rate; SV, stroke volume; SW, stroke work; V_E_, ventilation; V̇O_2_, oxygen uptake; LAC, blood lactate concentration; n^2^_p_, partial eta-squared

*Significant differences, the values are given as the means and standard deviations for comparison at the 125 W stage and maximum output (post-test)

**Fig. 2:**
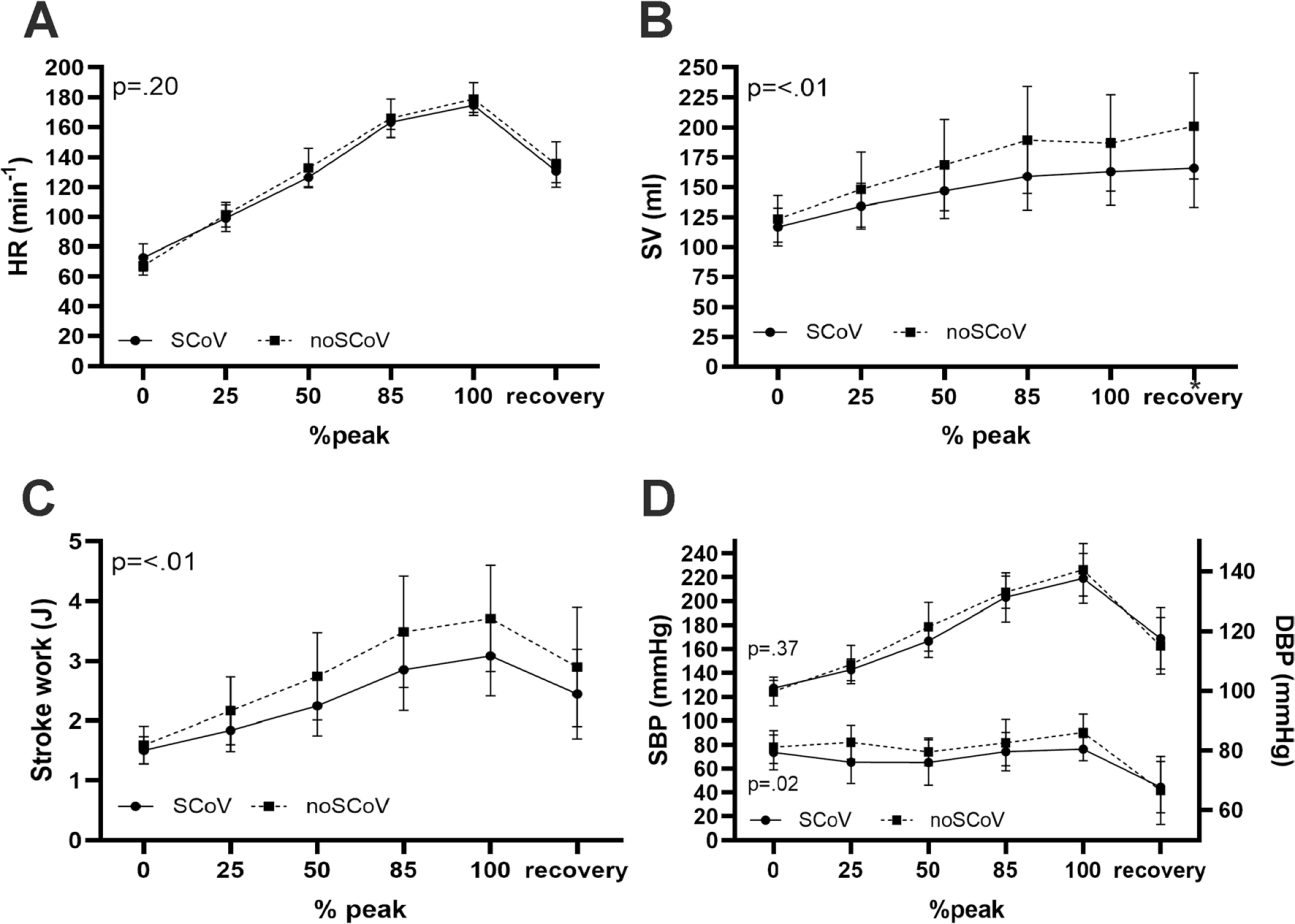
Two-way ANOVA Cardio-circulatory hemodynamic parameters at post-test T2: **A** Heart rate (group means: 127.7 vs. 13.2 bpm); **B** Stroke volume (group means: 147.5 vs. 169.5 ml); **C** Stroke work (group means: 2.3 vs. 2.8 J); **D** Systolic blood pressure (group means: 171 vs. 174 mmHg) and Diastolic blood pressure (group means: 76.6 vs. 79.8 mmHg) [*Significant differences in group comparison by Bonferroni test]

**Fig. 3:**
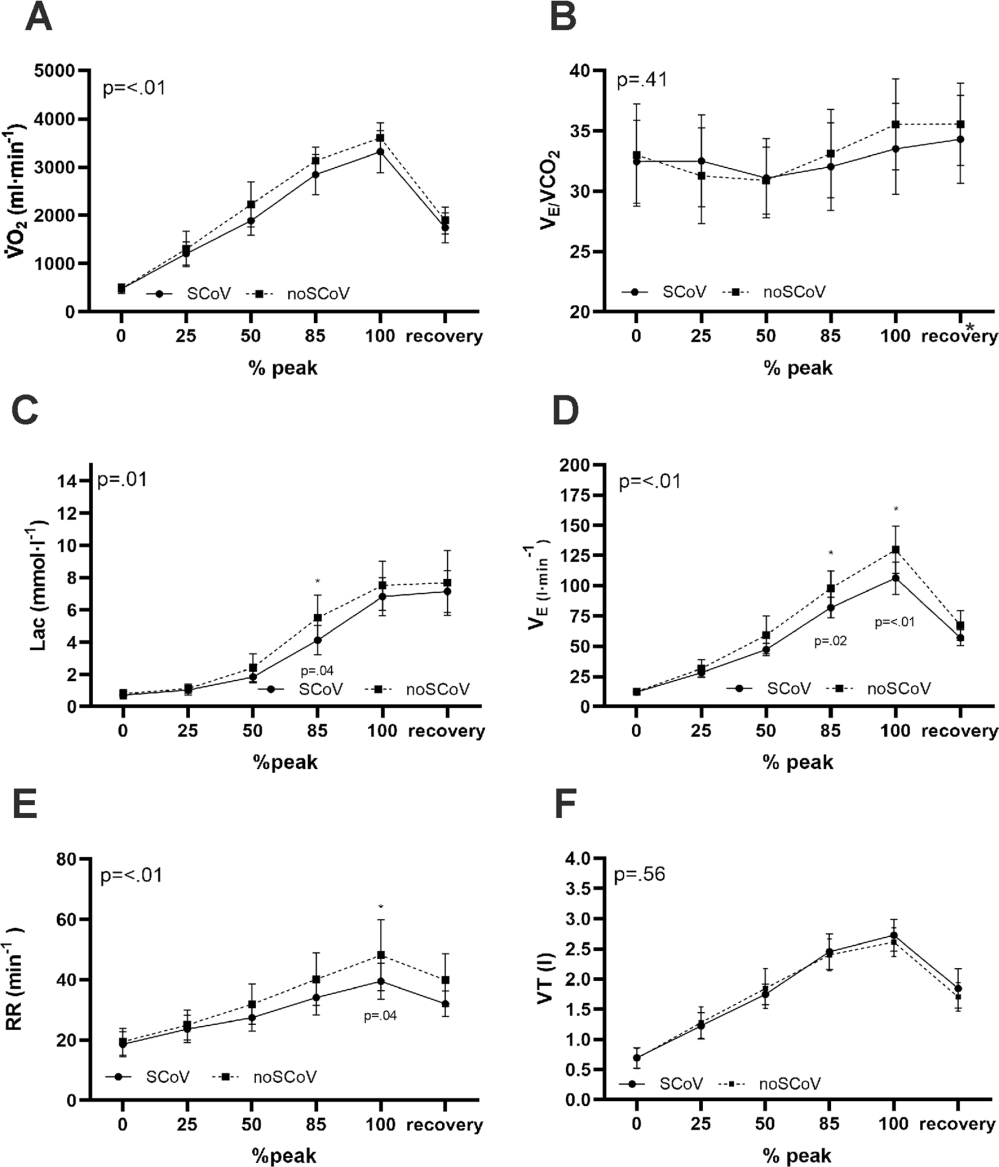
Tow way ANOVA Ventilatory-metabolic hemodynamic parameters at post-test T2: **A** Oxygen uptake (group means: 1912 vs. 2106 ml); **B** Ventilatory equivalent for carbon dioxide (group means: 32.6 vs. 33.2 l/ml); **C** Blood lactate concentration (group means: 3.6 vs. 4.2 mmol/l); **D** Minute Ventilation (group means: 55.5 vs. 66.4 l/min); **E** Respiratory rate (group means: 29.2 vs. 34.1 bpm); **F** Tidal volume (group means: 1.8 vs. 1.8 l/ml) [*Significant differences in group comparison by Bonferroni test]

To compare cardiopulmonary and circulatory mean values across rest, exercise, and post-exercise periods (independent groups with repeated measurement time points during exercise; Figs. 2, 3) a two-way ANOVA with Bonferroni's multiple comparison test for group differences was used. To assess the probability of occurrence of premature ventricular complexes (PVCs), the odds ratio was used.

## Results

The pre-exercise test (T1) revealed no differences in maximum power output between groups (SCoV 306.7 ± 38.6 W vs. noScoV 300.0 ± 32.7 W, *p* = 0.70).

### Differences within groups between the pre-test (T1) and post-test (T2)

Table 2 shows changes within groups between the pre-test (T1) and post-test (T2).

Table 2 shows that the maximum power output changed significantly in the SCoV group compared to the noSCoV group between the test time points. The SCoV group showed significantly higher hsCRP levels in the post-test (T2) than in the pre-test (T2), but within the clinically safe reference range.

### Results for resting measurements (DLCO, 24 h blood pressure, echocardiography) in the post-test (T2)

Only 16 subjects were able to complete the DLCO measurements and 24-h long-term blood pressure measurements. Table 3 shows the group comparison for the resting parameters at the second measurement time point (post-test T2). The cardiac blood markers (NT-ProBNP and Troponin T) showed no group differences and fell within the reference range.

### Results for exercise measurements in the post-test (T2)

Table 4 shows the changes between groups at post-test (T2) at 125 W and at maximum power output. We identified no statistically significant differences between groups in maximum power output (SCoV 258.3 ± 34.7 W vs. noSCoV 285.0 ± 38.2 W, *p* = 0.14/n^2^_p_ = 0.13). Neither were there any significant differences in their RPE (SCoV 9.11 ± 0.78 vs. noSCoV 9.56 ± 0.53, *p* = 0.31/n^2^_p_ = 0.11), nor any differences in the parameter respiratory quotient (RQ) under maximum stress (SCoV 0.96 ± 0.07 vs. noSCoV 1.0 ± 0.08, *p* = 0.13/n^2^_p_ = 0.14).

Figure 2 shows cardio-circulatory hemodynamic parameters, Fig. 3 ventilatory-metabolic hemodynamic parameters.

The Odds-ratio of PVCs after a SARS-CoV-2 infection is 1.6 (with 95% CI of [0.24–10.81]) cases higher than without having been infected. None of the SCoV group players showed PVCs in the ECG in the pre-test (T1), but 4 players showed VES under stress in the post-test (T2). In the noSCoV group, 1 individual showed PVCs under stress in the first test (T1) and 3 individuals showed PVCs under stress in the second test (T2).

## Discussion

The SARS-CoV-2-infected athletes exhibited longitudinally significantly reduced maximum power output, unlike the non-infected group. The infected group’s V̇O_2_ levels during incremental exercise were thus lower than the non-infected group’s. Our study could not identify any pulmonary causes for the reduced performance between the test time points. The SCoV group’s greater loss of exercise performance in ergometer testing at the second time point appears attributable to a lower stroke volume with unchanged heart rate kinetics. No differences in cardiopulmonary parameters were detectable under resting conditions.

### Pulmonary function

Our results show that the pulmonary function parameters (VC, FEV1, PEF) did not change between test days in either group. There were also no differences in the R_AW_ parameter on the post-test (T2). Our data reveal, as does the literature, no evidence of obstructive ventilation disorder [10, 13, 22, 23]. Moreover, we detected no differences in DLCO and DLCO/VA between groups with and without a reported SARS CoV-2 infection in the post-test (T2). Komici et al. [24] showed that young competitive athletes experiencing mild symptoms after SARS-CoV-2 infection did not present an impaired performance or cardiopulmonary parameters, with the exception of decreased FEV1. The post-term diffusion capacity (DLCO) effects of a SARS CoV-2 infection taking a mild or asymptomatic course in healthy young competitive athletes have not been described until now. Huang et al. and Blanco et al. described the occurrence of diffusion limitations depending on the disease course’s severity [25, 26], which could explain why our athletes who were predominantly asymptomatic demonstrated no DLCO or DLCO/VA restrictions. We observed no changes in resting lung function across different time points—a finding consistent with other studies of mild COVID-19 cases in competitive athletes [13, 22, 23].

Mitrani et al. [12] demonstrated in competitive athletes that in some cases, proven myocardial involvement can increase the respiratory efficiency parameter (V_E_/VCO_2_). The present data show no differences between groups with respect to V_E_/VCO_2_ kinetics at rest and during exercise. Nevertheless, the SCoV group’s V_E_ and RR were significantly reduced at a high ergometric workload. There is evidence that V_E_ during exercise and its innervation is due to intense muscular stimulation [27–30]. The within-group differences between the pre-test (T1) and post-test (T2) showed that the SCoV group’s maximum power and PRE decreased significantly in contrast to the noSCoV group. At the same wattage, PRE, and VO_2_ in the post-test (Table 4), we noted physiologically and noticeably lower V_E_ during moderate exercise in our group comparison. The post-test (T2) group comparison showed significantly reduced blood lactate concentration kinetics in the SCoV group under relative submaximum conditions (Fig. 3), thus might suggesting that the lower feedback from mechanosensitive and metabosensitive afferents of skeletal muscle (of group III/IV) could explain the lower V_E_ values under a high load [27, 29, 30].

The present data at rest and during exercise yield no evidence that the infected athletes’ apparent decreased exercise capacity is due to pulmonary restrictions caused by the infection [10, 13, 22, 23].

### Cardiac circulation function

We detected no clinical abnormalities in the two groups’ cardiac laboratory parameters (high-sensitivity C-reactive protein, high sensitive Troponin T, NT-proBNP) in either the pre-test or post-test. Both groups showed pronounced SBP above the reference value at the 24-h blood pressure measurement at post-test, and did not differ (Table 3). Echocardiography showed unchanged EF% values over time and no difference between groups. The GLS value showed a significant reduction from pre-test (T1) to post-test (T2) in both groups (Table 2). However, there were no differences between the two groups in the post-test comparison—results that confirm the latest state of knowledge [5, 8, 12, 13, 31].

Studies show that no ECG, echocardiographic, or laboratory abnormalities were detected in SARS-CoV-2-infected competitive athletes, but CMR follow-ups revealed subclinical or evidence of subclinical myocarditis [5, 8]. Małek et al. [8] showed that 19% of young competitive athletes exhibit abnormalities on cardiac magnetic resonance imaging (CMR), but no abnormal electrocardiographic anomalies, troponin elevations, or signs of acute clinical myocarditis after a mild SARS-CoV-2 infection. In the study by Daniels et al. [5], 20 of 37 athletes presented detectable signs of subclinical myocarditis in the CMR but no symptoms, no abnormal ECG findings, unremarkable echocardiographic findings, and no troponin elevations. In this context, myocarditis can be classified in three phases: (1) active inflammatory infection phase (clinical myocarditis with heart symptoms before or at the time of cardiac examination), (2) immune response to infection and resulting scar tissue and/or cardiomyopathy (subclinically probable myocarditis without cardiac symptoms, but with abnormal ECG, echocardiogram, or troponin), (3) no clinical abnormalities or abnormal CMR findings (subclinically possible myocarditis without cardiac symptoms, and no abnormal ECG, echocardiogram, or troponin findings) [5, 8]. Mitrani et al. [12] showed in their large-scale study that 2.9% of the athletes presented myocardial involvement. They demonstrated that left ventricular ejection fraction and GLS were similar in athletes with and without myocardial involvement. During exercise testing, no differences in V̇O_2_ max were observed in athletes with myocardial involvement, but the percentage of premature ventricular complexes (PVCs) occurring was significantly increased [12]. Nevertheless, these studies demonstrate that even mild SARS-CoV-2 infection can be clinically relevant and difficult to diagnose without CMR [5, 8] Consistent with these observations, the SCoV group’s odds ratio for the PVCs under stress was 1.6 [10, 12]. We took no CMR measurements. Recent evidence suggests that the myocardium also contains a high concentration of ACE2 receptors, so that the known binding of SARS CoV-2 virus to these may trigger a direct pathophysiological chain of cardiac events [3, 32].

However, our exercise results show that the SCoV group’s V̇O_2_ course during exercise was lower. Fikenzer et al. [13] obtained similar results where their infected group of competitive handball players revealed a similarly weaker ergometric performance in the pre-post comparison, while their V̇O_2_ parameters were also lower. They observed a decreased oxygen pulse in infected elite handball players in an incremental exercise test and suggested that this was related to a reduction in SV. Cardiac resonance imaging performed in their investigation showed no statistically significant differences between infected and uninfected groups [13]. Nevertheless, our study shows that when comparing our groups’ post-tests, SV and SW kinetics during exercise were significantly lower in the SCoV group (Fig. 2). Exercise tachycardia is characterized by a decrease in end-diastolic volume despite a progressive increase in filling pressure, so that stroke volume must be maintained by a decrease in end-systolic volume [33]. With the same HR kinetics but lower SV, an effect on left ventricular contractility after SARS-CoV-2 infection seems possible. Despite noticeably lower maximum wattage (T2), the the SCoV group’s heart rate did not change in. Our data thus suggest a hyperproportional HR regulation at the same exercise load as a sign of deconditioning [13].

The SCoV group’s DBP parameter showed lower values than in the noScoV group during exercise (Fig. 3, Table 4). This could be due to lower absolute performance and/or lower SV [34]. In contrast, there were no statistically significant group differences in maximum wattage, PRE and RQ when comparing the post-test (Table 4). However, the difference in power between the infected and non-infected group was 7 W in T1 and 27 W in T2. From a performance-physiology perspective, this looks like a significant difference in performance. A recent study shows that mild courses of covid-19 can lead to persistent cardiac symptoms with partial inflammatory cardiac involvement in previously healthy individuals [14]. The present study did not use MRI screening but found no evidence of echocardiographic or laboratory abnormalities. Nevertheless, significantly low cardiac functional performance was evident. In conclusion, the present results do not reveal pulmonary [10, 13, 22, 23], laboratory chemistry, or echocardiographic abnormalities [5, 8, 13]. However, our group comparison (T2) demonstrated an abnormality in the occurrence of PVCs [10, 12] and reduced V̇O_2_ kinetics during exercise [13] in the SCoV group. The reduced SV and lower SW despite the same HR, PRE, and lactate values under identical wattage conditions (T2) may indicate potential cardiac deconditioning, or limitations attributable to a deficit in conditioning.

### Limitation

As our sample size is small and only male participants were enrolled, the generalizability of our results is limited. Our analysis of the PVC parameter using the odds ratio should be considered critically because of our small subject cohort. Nevertheless, we consider our findings to be clinically relevant [35]. However, this is the first trial to present longitudinal results of exercise parameters in a cohort study with a group of SARS-CoV-2-infected and non-infected athletes. We are unaware of any similar study in the literature involving a larger number of subjects. The examinations in 2020 (T1) were conducted without ergospirometry, impedance cardiography and diffusion measurements, since the examinations had to be sports-suitable. A comparison of these parameters between t1 and t2 is therefore not possible. Cardiac parameters in 2021 obtained via impedance cardiography may be overestimated using absolute values [36]. The time point of infection cannot be specified due to a partially blind Coronavirus disease course.

## Conclusion

In conclusion: handball players infected with SARS-CoV-2 did not have pneumonia, and their disease course was predominantly asymptomatic; they also displayed no impairments in diffusion or pulmonary function compared to our uninfected control group. However, the SCoV group’s maximum power, but not their heart rate was significantly lower than that of the control group compared to baseline measurements. The present exercise results show significantly reduced V̇O_2_-, SV-kinetics, and a tendency toward lower wattage at comparable heart rates, as well as an increased incidence of PVCs in athletes who had a SARS-CoV-2 infection. Our data suggest virus-induced deconditioning leading to reduced cardiac efficiency. Our findings demonstrate the importance of cardiac screening before resuming sport activities after surviving a SARS-CoV-2 infection. The underlying mechanisms of deconditioning after SARS-CoV-2 infection are not completely understood at this time, thus warranting further research.

## Data Availability

The datasets generated during the present study can be obtained from the corresponding author on reasonable request. The trail results will be communicated via publications.

## Abbreviations

BP: Blood pressure
CO: Cardiac output
CO_M_: Carbon monoxide
DL_CO_: Lung diffusing capacity for carbon monoxide
DL_CO_/V_A_: Lung diffusion capacity divided by the alveolar volume capacity for carbon monoxide
EF: Ejection faction
GLS: Global longitudinal strains
SW: Stroke work
HR: Heart rate
LAC: Blood lactate concentration
DFP: Pressure difference product
R_AW_: Airway resistance
PVCs: Premature ventricular complexes
RPE: Rating of perceived exertion
RPP: Rate pressure product
RR: Respiratory rate
SV: Stroke volume
TPR: Total peripheral resistance
VC: Vital capacity
VE: Minute ventilation
V̇CO_2_: Carbon dioxide production
V̇O_2_: Oxygen uptake
VT: Tidal volume
